# Supplementary material for: Comparison of FDA accelerated vs regular pathway approvals for lung cancer treatments between 2006 and 2018
Source: PLoS One. 2020 Jul 24;15(7):e0236345. doi: 10.1371/journal.pone.0236345 (PMC7380631; doi:10.1371/journal.pone.0236345)
Supplement: S1 Table — (DOCX) [file pone.0236345.s001.docx]

S1 Table: Details of approvals exclusion and justification

| Generic name | Approval date | Data source | Reason for exclusion |
| --- | --- | --- | --- |
| crizotinib | Nov, 2013 | <http://wayback.archive-it.org/7993/20170111231705/http://www.fda.gov/Drugs/InformationOnDrugs/ApprovedDrugs/ucm376058.htm> | Conversion from accelerated approval to regular approval |
| nivolumab | Sept, 2016 | <https://www.fda.gov/drugs/resources-information-approved-drugs/modification-dosage-regimen-nivolumab> | Dosage modification |
| erlotinib | Oct, 2016 | <http://wayback.archive-it.org/7993/20170111231550/http://www.fda.gov/Drugs/InformationOnDrugs/ApprovedDrugs/ucm525739.htm> | Label alteration regarding EGFR just from exon 19 and 21 to EGFR in general in 2nd line |
| pembrolizumab | Oct, 2016 | <http://wayback.archive-it.org/7993/20170111231548/http://www.fda.gov/Drugs/InformationOnDrugs/ApprovedDrugs/ucm526430.htm> | Conversion from accelerated approval to regular approval |
| osimertinib | Mar, 2017 | <https://www.fda.gov/drugs/resources-information-approved-drugs/osimertinib-tagrisso> | Conversion from accelerated approval to regular approval |
| ceritinib | May, 2017 | <https://www.fda.gov/drugs/resources-information-approved-drugs/fda-broadens-ceritinib-indication-previously-untreated-alk-positive-metastatic-nsclc> | Conversion from accelerated approval to regular approval |
| alectinib | Nov, 2017 | <https://www.fda.gov/drugs/resources-information-approved-drugs/alectinib-approved-alk-positive-metastatic-non-small-cell-lung-cancer-nsclc> | Conversion from accelerated approval to regular approval |
| pembrolizumab | Aug, 2018 | <https://www.fda.gov/drugs/resources-information-approved-drugs/fda-grants-regular-approval-pembrolizumab-combination-chemotherapy-first-line-treatment-metastatic> | Conversion from accelerated approval to regular approval |
