# Supplementary material for: Comparison of FDA accelerated vs regular pathway approvals for lung cancer treatments between 2006 and 2018
Source: PLoS One. 2020 Jul 24;15(7):e0236345. doi: 10.1371/journal.pone.0236345 (PMC7380631; doi:10.1371/journal.pone.0236345)
Supplement: S2 Table — (DOCX) [file pone.0236345.s002.docx]

S2 Table: Details of the control selection and risk ratio calculation.
