# Supplementary material for: Comparison of FDA accelerated vs regular pathway approvals for lung cancer treatments between 2006 and 2018
Source: PLoS One. 2020 Jul 24;15(7):e0236345. doi: 10.1371/journal.pone.0236345 (PMC7380631; doi:10.1371/journal.pone.0236345)
Supplement: Attachment — Submitted filename: Letter PLOS ONE - Review.docx [file pone.0236345.s003.docx]

**May 30th, 2020**

Editor and Reviewers of

**PLOS ONE**

Subject: Review of the Original Article “Comparison of FDA Accelerated vs Regular Pathway Approvals for Lung Cancer Treatments between 2006 and 2018”

*.*

*Dear Editor, Dear Reviewers,
Thank for your all useful comments on our manuscript. We have addressed them in the current version and we believe the manuscript has been improved. All changes made to the original version are highlighted in a marked up copy of our manuscript.
Please find our responses below.
Yours sincerely,
Tatiane Ribeiro*

Reviewer #1: Authors have compared FDA accelerated vs regular pathways approvals and breakthrough therapy designations for lung cancer treatment between 2006 and 2018. The authors concluded that fater approvals are in the majority full of uncertainties which should be viewed with caution and the patient have to be communicated to allow shared decision making.
The manuscript is very well written and informative. It could be accepted for publication.

*Thanks. Nothing to add.*

Reviewer #2: Ribeiro and co. compared the approved accelerated and approved regular clinical studies. They stated that the “larger effect size” is not different from the accelerated one and the regular approval for FDA lung cancer approvals between 2006 and 2018.
This suggestion is very important, but this statement should be better critically analysed.

In particular the final conclusion could be biased, as also suggested by the authors, from the surrogated end points used in regular and accelerated approved studies. The Authors reported the GRADE method, but this method is based on the quality of evidences and substantial end point , i.e. the overall survival. Even if the Authors have evidenced several limitations of the analyses ( surrogate markers, lack of control group,…) they still conclude that there are not differences in the “larger effect size”.

*Thanks for the comments, we clarified these aspects in the conclusion.*

Specific comments
• The effect size should be better explained and an explicative table, reporting statistics for all the parameter considered, could be useful

*We are grateful for the reviewer’s comments, we already information regarding table 1, table 2 and supplemental material that explain raw data. In order to add information regarding effect sizes, according to reviewers suggestion we opted to create Graphs (Graph 1-4) to demonstrate effect sizes differences in Accelerated approval pathway and Breakthrough therapy designation.*

• With regard to the study design, have the statistic power and the sample size been considered?

*We included this information at the Methods session: “The studies were primarily descriptive, and we included all data available for lung cancer treatment in the period (2006-2018).”*

*Thus, as we included all the information available of studies used to approval for lung cancer treatment, power calculation is not necessary.*
